# Prognostic Relevance of Urinary Bladder Cancer Susceptibility Loci

**DOI:** 10.1371/journal.pone.0089164

**Published:** 2014-02-25

**Authors:** Anne J. Grotenhuis, Aleksandra M. Dudek, Gerald W. Verhaegh, J. Alfred Witjes, Katja K. Aben, Saskia L. van der Marel, Sita H. Vermeulen, Lambertus A. Kiemeney

**Affiliations:** 1 Department for Health Evidence, Radboud University Medical Center, Nijmegen, The Netherlands; 2 Department of Urology, Radboud University Medical Center, Nijmegen, The Netherlands; 3 Comprehensive Cancer Center The Netherlands, Utrecht, The Netherlands; 4 Department of Human Genetics, Radboud University Medical Center, Nijmegen, The Netherlands; University of British Columbia, Canada

## Abstract

In the last few years, susceptibility loci have been identified for urinary bladder cancer (UBC) through candidate-gene and genome-wide association studies. Prognostic relevance of most of these loci is yet unknown. In this study, we used data of the Nijmegen Bladder Cancer Study (NBCS) to perform a comprehensive evaluation of the prognostic relevance of all confirmed UBC susceptibility loci. Detailed clinical data concerning diagnosis, stage, treatment, and disease course of a population-based series of 1,602 UBC patients were collected retrospectively based on a medical file survey. Kaplan-Meier survival analyses and Cox proportional hazard regression were performed, and log-rank tests calculated, to evaluate the association between 12 confirmed UBC susceptibility variants and recurrence and progression in non-muscle invasive bladder cancer (NMIBC) patients. Among muscle-invasive or metastatic bladder cancer (MIBC) patients, association of these variants with overall survival was tested. Subgroup analyses by tumor aggressiveness and smoking status were performed in NMIBC patients. In the overall NMIBC group (n = 1,269), a statistically significant association between rs9642880 at 8q24 and risk of progression was observed (GT vs. TT: HR = 1.08 (95% CI: 0.76–1.54), GG vs. TT: HR = 1.81 (95% CI: 1.23–2.66), P for trend = 2.6×10^−3^). In subgroup analyses, several other variants showed suggestive, though non-significant, prognostic relevance for recurrence and progression in NMIBC and survival in MIBC. This study provides suggestive evidence that genetic loci involved in UBC etiology may influence disease prognosis. Elucidation of the causal variant(s) could further our understanding of the mechanism of disease, could point to new therapeutic targets, and might aid in improvement of prognostic tools.

## Introduction

Urinary bladder cancer (UBC) is a heterogeneous disease with respect to its prognosis. The available prognostic tools that are based on clinicopathological variables, such as the European Organisation for Research and Treatment of Cancer (EORTC) risk tables and the Club Urológico Español de Tratamiento Oncológico (CUETO) scoring model, have insufficient discriminative ability to accurately predict the risk of disease recurrence and progression at the level of the individual patient. [1] The same holds for scores that include molecular markers. [2], [3] Additional or better markers are clearly needed for personalized healthcare. There is growing evidence for a role of (germline) genetic polymorphisms in disease prognosis and treatment response. [4]–[7] Identification of such genetic variants may lead to improvement of disease outcome prediction in UBC patients. Discovery of such variants might also provide clues about the underlying mechanism of urothelial carcinogenesis and cancer progression, and thereby point the way to new therapeutic targets.

Sufficiently powered and well-designed studies into the prognostic and predictive value of germline genetic polymorphisms in UBC are rare. Chen *et al.* identified and (externally) validated the influence of genetic variation in the sonic hedgehog pathway (*i.e.*, rs1233560 in *SHH* (sonic hedgehog) and rs11685068 in *GLI2* (GLI family zinc finger 2)) on the risk of recurrence after transurethral resection of the tumor (TURT) in non-muscle invasive bladder cancer (NMIBC) patients. [8] The same research group discovered association of a polymorphism in one of the microRNA biogenesis genes (*i.e.,* rs197412 in *DDX20* (DEAD (Asp-Glu-Ala-Asp) box polypeptide 20)) with disease recurrence in the same UBC subgroup, which could be replicated in an additional NMIBC patient series. [9] The remainder of the published candidate-gene surveys for UBC prognosis is of small size and still awaits independent replications to exclude false-positive findings. [10]–[12] Genome-wide association studies (GWAS) into UBC prognosis are still lacking. For UBC *susceptibility*, the shift to an agnostic GWAS approach has led to the successful identification of several novel, established genetic polymorphisms. [13] For several cancer types, including colorectal, pancreatic, breast, lung, and prostate cancer, it has been shown that GWAS-identified susceptibility variants also have prognostic relevance. [14]–[20] Indeed, in one of our GWAS for UBC susceptibility, we found that the T allele of the identified risk variant rs798766 (*TACC3/FGFR3* (transforming, acidic coiled-coil containing protein 3/fibroblast growth factor receptor 3) locus) is associated with a higher risk of recurrence, specifically among patients with low-grade Ta tumors. [21] Here, we comprehensively evaluate the prognostic relevance of 12 variants at 11 (extensively) replicated UBC susceptibility loci. Our study indicates that there is overlap in genetic variants underlying UBC etiology and prognosis.

## Materials and Methods

### Ethics statement

The study was conducted according to the principles expressed in the declaration of Helsinki. All participants gave written informed consent and the study was approved by the Institutional Review Board of the Radboud university medical center, Nijmegen, the Netherlands.

### Patient population

This study was performed in 1,602 patients with primary UBC from the Nijmegen Bladder Cancer Study (NBCS). The NBCS served as the Dutch discovery population in the UBC GWAS led by Radboud university medical center and deCODE Genetics (Reykjavik, Iceland). The NBCS has been described in detail before. [13] Patients with a previous or simultaneous (within three months) diagnosis of upper urinary tract cancer, based on information from the Netherlands Cancer Registry (NCR), were excluded. Detailed clinical data concerning diagnosis, stage, treatment, and disease course (tumor recurrence and progression) was collected retrospectively by a medical file survey. Based on stage and histological grade, all NMIBC patients were classified with regard to tumor aggressiveness (*i.e.*, risk of progression). Subjects with low risk of progression were defined as those having TNM stage Ta in combination with WHO 1973 differentiation grade 1 or 2, WHO/ISUP 2004 low grade, or Malmström (modified Bergkvist) grade 1 or 2a. All other patients were classified as having tumors with high risk of progression (stage CIS ór T1 ór WHO 1973 grade 3, WHO/ISUP 2004 high grade, or Malmström (modified Bergkvist) grade 2b or 3). Self-reported data on smoking status was available based on a lifestyle questionnaire filled out by the participants at study inclusion.

### Selection of variants and genotyping

The ten UBC susceptibility single-nucleotide polymorphisms (SNPs) that were identified through GWAS for UBC risk and replicated in at least one independent population, were selected for this study (see Table 1 for details). All of these SNPs were genotyped via the Illumina HumanCNV370-Duo BeadChip, except for rs2736098 that was genotyped by a single-SNP Centaurus (Nanogen) assay. [22] The genome-wide genotyping of SNPs in the NBCS, and related quality control (QC) procedures were described in detail before. [13] The concordance rate between (UBC susceptibility) SNP genotypes measured using the Illumina platform and those derived from a single-SNP Centaurus (Nanogen) assay was previously found to be >99.5%. [13], [21], [23]

**Table 1:** Extensively replicated germline UBC susceptibility loci.

Locus	Gene region	SNP	Risk allele^a^	Allelic OR^a^	Risk allele frequency^b^	Study type	Reference
8p22	*NAT2*	-	slow acetylator	1.4	0.56	Candidate-gene	[45]
1p13.3	*GSTM1*	-	null	1.5	0.51	Candidate-gene	[45]
8q24.21	*MYC*	rs9642880	T	1.22	0.45	GWAS	[23]
3q28	*TP63*	rs710521	A	1.19	0.73	GWAS	[23]
5p15.33	*TERT*	rs2736098	A	1.16	0.26	GWAS	[22]
5p15.33	*CLPTM1L*	rs401681	C	1.12	0.54	GWAS	[22]
8q24.3	*PSCA*	rs2294008	T	1.15	0.46	GWAS	[46]
4p16.3	*TACC3-FGFR3*	rs798766	T	1.24	0.19	GWAS	[21]
22q13.1	*CBX6, APOBEC3A*	rs1014971	T	1.14	0.62	GWAS	[47]
19q12	*CCNE1*	rs8102137	C	1.13	0.33	GWAS	[47]
2q37.1	*UGT1A*	rs11892031	A	1.19	0.92	GWAS	[47]
18q12.3	*SLC14A1*	rs1058396	G	1.14	0.50	GWAS	[13]

OR: odds ratio; *TP63*: tumor protein p63; *TERT*: telomerase reverse transcriptase; *CLPTM1L*: CLPTM1-like; *PSCA*: prostate stem cell antigen; *CBX6*: chromobox homolog 6; *APOBEC3A*: apolipoprotein B mRNA editing enzyme, catalytic polypeptide-like 3A; *CCNE1*: cyclin E1; *SLC14A1*: solute carrier family 14 (urea transporter), member 1

a for *NAT2* and *GSTM1* these do not refer to the risk allele but to the risk genotype;

b risk allele frequency among controls as published in candidate-gene study/GWAS paper

In addition, deletion of the glutathione S-transferase mu 1 (*GSTM1*) gene (null genotype) and a tag SNP for the N-acetyltransferase 2 (*NAT2*) slow acetylation phenotype (rs1495741) [24], both with an established influence on UBC risk based on (meta-analysis of) candidate-gene studies, were included in this study (see Table 1). *GSTM1* copy number variation (CNV) status was determined by an Applied Biosystems TaqMan Copy Number assay (Assay ID: Hs02575461_cn). The *NAT2* tagSNP was genotyped through the Illumina HumanCNV370-Duo BeadChip.

As we realize that the 10 GWAS-identified genetic variants evaluated are not necessarily the causal variants, we also evaluated genetic variants in a 200 kb region centered on each of the susceptibility SNPs in relation to each of the prognostic endpoints. For this, we used genome-wide measured and imputed SNP data. Imputation was performed using the 1000 Genomes low-coverage pilot haplotypes (released June 2010, 120 chromosomes) and the HapMap3 haplotypes (released February 2009, 1920 chromosomes) as a combined reference panel. [13] We thereby automatically included the two UBC susceptibility variants (*i.e.*, rs2978974 (8q24.3) and rs17863783 (2q37.1)) that were identified in previously published finemapping efforts. [25], [26]

### Outcome definition

In the NMIBC subgroup, the association of the 12 variants with the prognostic endpoints recurrence-free survival (RFS) and progression-free survival (PFS) was investigated. *Date of first recurrence* was defined as date of histological confirmation of a newly found bladder or prostatic urethra tumor following at least one tumor-negative follow-up cystoscopy or two surgical resection sessions for the primary tumor. *Date of first progression* was defined as date of first occurrence of grade progression, stage progression, local and/or distant metastasis, and/or cystectomy for therapy-resistant (‘uncontrollable’) disease. See Text S1 for a more detailed description of the prognostic endpoint definitions. NMIBC patients who were treated with an immediate radical cystectomy after primary diagnosis were considered not at risk of (intravesical) recurrence, and therefore excluded from further analyses. In case of no recurrence/progression, follow-up was censored at the last date of urological check-up. Only the first 5 years after the primary NMIBC diagnosis were considered in the analyses in order to focus on the most clinically relevant period for those prognostic endpoints, and also in order to reduce the effect of competing risks (especially for older patients). RFS and PFS were defined as the time period between date of the initial TURT and date of first event (recurrence or progression, respectively), date of censoring, or date of five-year follow-up, whichever came first.

In the subgroup of muscle-invasive (≥T2) or metastatic bladder cancer (MIBC) patients, the association between the 12 variants and overall survival (OS) was evaluated. For this purpose, information on vital status was retrieved via the NCR through record linkage to the nationwide Dutch Municipal Personal Records Database. If patients were still alive at December 31^st^, 2011, follow-up was censored at this date. Again, follow-up time considered was restricted to the first five years after diagnosis. OS was defined as the time period between date of the initial TURT and date of death (of all causes), date of censoring, or date of five-year follow-up, whichever came first.

### Statistical analysis

Kaplan-Meier survival and Cox proportional hazard regression analyses were performed, and log-rank tests calculated, to evaluate the association between the 12 variants and the above-mentioned prognostic endpoints. Multivariable Cox regression analysis was used to adjust the hazard ratio (HR) for the effect of treatment in NMIBC patients, and for extended/metastasized (*i.e.*, primary stage T4(b) ór any T with N+/N≥1 and/or M1) versus localized disease (*i.e*., primary stage T2-T4a with N0/NX and M0/MX) at diagnosis in MIBC patients. To evaluate subgroup-specific effects, a stratified analysis according to tumor aggressiveness of NMIBC (*i.e.*, low vs. high risk of progression) and smoking status (*i.e.*, never vs. ever cigarette smoking) was performed.

The association with disease prognosis was evaluated based on a genotypic model with the homozygous genotype of the most common (major) allele (based on our data) assigned as the reference category. For *GSTM1* we studied the association for patients with the null genotype compared to patients with at least one copy of the gene present, and for *NAT2* we evaluated the association of slow acetylators (rs1495741: AA) compared to intermediate/rapid acetylators (AG/GG). The Bonferroni correction was applied to adjust the statistical significance threshold for the 12 tested variants (alpha  =  0.05/12  =  4×10^−3^) (with P-values derived based on a (two-sided) trend test). At this alpha level and assuming a risk allele frequency of 0.20, our study had 80% power to detect a HR greater than 1.35 and 1.89 for recurrence (five-year risk: 50%), and a minimum HR of 1.57 and 2.36 for progression (five-year risk: 20%), according to a dominant and recessive mode of inheritance, respectively (IBM SPSS SamplePower release 3.01).

In addition to the single-SNP analyses, we evaluated the cumulative prognostic value of the UBC susceptibility variants by testing association with the genetic risk score, *i.e.*, total sum of the number of UBC risk alleles (0,1,2) for each SNP among individuals successfully genotyped for all 12 variants. For *NAT2* and *GSTM1*, we counted the risk genotype (0,1). Cumulative (additive) association with RFS and PFS among NMIBC patients was tested by including this genetic risk score as continuous (independent) variable in a Cox regression model (with adjustment for treatment type). Statistical analyses were performed using IBM SPSS Statistics for Windows 20 (IBM Corp., Armonk, NY, USA).

Association parameters for the imputed SNP variants surrounding the GWAS risk SNPs (according to an additive inheritance model) were obtained by Cox proportional hazards regression analyses performed with ProbABEL v0.1-3 from the GenABEL suite of programs. [27] The Cox proportional hazards model (*pacoxph* function) implemented in the ProbABEL-package makes use of the source code of the R package “survival” as implemented by T. Lumley. Regional association plots were drawn using LocusZoom software. [28]

## Results

### Non-muscle invasive bladder cancer (NMIBC)

Among the total study population of 1,602 UBC patients, 1,327 were diagnosed with NMIBC (stages Ta, T1, CIS). Thirty patients were excluded because they were previously or simultaneously diagnosed with cancer of the upper urinary tract. In addition, nine patients were excluded because the recurrence and progression status could not be validly assessed based on the medical file review. Finally, we excluded 19 NMIBC patients who had an immediate radical cystectomy (see Table S1 for patient and tumor characteristics). The median time between date of the initial TURT and date of the last urological check-up visit of the remaining 1,269 NMIBC patients was 5.3 (interquartile range (IQR): 3.7–8.7) years. Demographic and clinicopathological characteristics of both the NMIBC and MIBC group are shown in Table 2.

**Table 2:** Demographic and clinicopathological characteristics of included NMIBC and MIBC patients

N (%)		NMIBC	MIBC
		N = 1,269	N = 273
Male gender		1,065 (84)	196 (72)
Median age (range)		64 (25–92)	64 (27–93)
Smoking status	Never cigarette smoker	189 (15)	30 (11)
	Ever cigarette smoker	887 (70)	165 (60)
	Unknown	193 (15)	78 (29)
Tumor stage	0a	866 (68)	-
	0is	50 (4)	-
	I	336 (26)	-
	II	-	136 (50)
	III	-	50 (18)
	IV	-	87 (32)
	Unknown	17 (1.3)	-
Tumor grade	Low grade	775 (61)	15 (5.5)
	High grade	481 (38)	236 (86)
	Unknown	13 (1.0)	22 (8.1)
Tumor aggressiveness	Low risk of progression	703 (55)	-
	High risk of progression	552 (44)	-
	Unknown	14 (1.1)	-
Tumor histology	UCC	1,257 (99)	240 (88)
	SCC	-	11 (4.0)
	AC	1 (0.1)	9 (3.3)
	Other	2 (0.2)	11 (4.0)
	Unknown	9 (0.7)	2 (0.7)
Tumor size	<3 cm	181 (14)	15 (5.5)
	≥3 cm	93 (7.3)	39 (14)
	Unknown	995 (78)	219 (80)
Tumor focality	Solitary	699 (55)	179 (66)
	Multifocal	490 (39)	68 (25)
	Unknown	80 (6.3)	26 (9.5)
Initial treatment NMIBC	TURT only (± one immediate p.o. i.v. CT instillation)	552 (44)	-
	TURT + adjuvant i.v. CT	392 (31)	-
	TURT + adjuvant i.v. IT	248 (20)	-
	TURT + both adjuvant i.v. CT and IT	26 (2.1)	-
	Other	3 (0.2)	-
	Unknown	48 (3.8)	-
Initial treatment MIBC^a^	Curative intent	-	186 (68)
	Palliative intent	-	87 (32)

UCC: urothelial cell carcinoma; SCC: squamous cell carcinoma; AC: adenocarcinoma; p.o.: post-operative; i.v.: intravesical; CT: chemotherapy; IT: immunotherapy

a curative intent corresponds to treatment of tumors of stage T2-T4a with N0/NX and M0/MX; palliative intent corresponds to treatment of tumors of stage T4(b) ór any T with N≥1/N+ and/or M1

#### Association of UBC susceptibility loci with disease recurrence

Median time at risk for recurrence of the 1,269 included NMIBC patients was 2.9 years. During the first 5 years after the primary UBC diagnosis, 601 (Kaplan-Meier 5-year risk: 51.3%) NMIBC patients experienced disease recurrence. None of the 12 genetic variants examined showed a statistically significant association with RFS at the Bonferroni-adjusted or nominal significance level (P<0.05) (see Table 3).

**Table 3:** Association of confirmed UBC susceptibility variants with NMIBC recurrence and progression

		Disease recurrence (N = 1,269)^a^			Disease progression (N = 1,269)^a^		
SNP/CNV	Genotype	N (n events)	HR (95% CI)	P trend	N (n events)	HR (95% CI)	P trend
rs9642880	TT	355 (180)	Ref.	0.98	355 (46)	Ref.	2.6×10^−3^
	GT	637 (281)	0.84 (0.70–1.01)		637 (88)	1.08 (0.76–1.54)	
	GG	269 (135)	1.03 (0.82–1.28)		269 (59)	1.81 (1.23–2.66)	
rs710521	AA	732 (357)	Ref.	0.23	732 (107)	Ref.	0.25
	AG	468 (212)	0.88 (0.75–1.05)		468 (74)	1.09 (0.81–1.46)	
	GG	68 (31)	0.94 (0.65–1.36)		68 (14)	1.42 (0.82–2.49)	
rs2294008^b^	CC	323 (144)	Ref.	0.16	323 (50)	Ref.	0.97
	CT	699 (332)	1.09 (0.90–1.33)		699 (107)	0.97 (0.69–1.36)	
	TT	246 (125)	1.19 (0.94–1.51)		246 (38)	1.00 (0.65–1.52)	
rs798766	CC	747 (339)	Ref.	0.12	747 (118)	Ref.	0.40
	CT	452 (229)	1.17 (0.99–1.39)		452 (69)	0.96 (0.71–1.29)	
	TT	69 (33)	1.10 (0.77–1.57)		69 (8)	0.69 (0.34–1.42)	
rs401681	CC	436 (212)	Ref.	0.76	436 (73)	Ref.	0.35
	CT	633 (293)	0.89 (0.74–1.06)		633 (94)	0.87 (0.64–1.18)	
	TT	199 (96)	1.02 (0.80–1.29)		199 (28)	0.84 (0.54–1.30)	
rs2736098	GG	482 (211)	Ref.	0.36	482 (60)	Ref.	0.12
	AG	408 (198)	1.12 (0.92–1.36)		408 (64)	1.28 (0.90–1.82)	
	AA	100 (46)	1.08 (0.78–1.48)		100 (17)	1.39 (0.81–2.39)	
rs11892031^c^	AA	1063 (502)	Ref.	0.56	1063 (160)	Ref.	0.44
	AC	201 (95)	1.01 (0.81–1.26)		201 (34)	1.15 (0.79–1.66)	
	CC	5 (4)	2.43 (0.91–6.50)		5 (1)	1.36 (0.19–9.71)	
rs8102137	TT	535 (252)	Ref.	0.67	535 (77)	Ref.	0.20
	CT	580 (273)	0.98 (0.83–1.17)		580 (88)	1.05 (0.77–1.42)	
	CC	146 (71)	1.10 (0.84–1.43)		146 (28)	1.40 (0.91–2.15)	
rs1014971	AA	564 (273)	Ref.	0.46	564 (81)	Ref.	0.43
	AG	585 (274)	0.95 (0.80–1.12)		585 (95)	1.15 (0.86–1.55)	
	GG	119 (54)	0.92 (0.68–1.23)		119 (19)	1.12 (0.68–1.84)	
rs1058396	GG	381 (170)	Ref.	0.22	381 (55)	Ref.	0.36
	AG	625 (296)	1.05 (0.87–1.27)		625 (93)	1.03 (0.74–1.43)	
	AA	262 (135)	1.16 (0.92–1.45)		262 (47)	1.21 (0.82–1.79)	
rs1495741^d^	GG/AG	457 (207)	Ref.	0.25	457 (71)	Ref.	1.00
	AA	811 (394)	1.10 (0.93–1.31)		811 (124)	1.00 (0.75–1.34)	
*GSTM1* deletion	+/+ and +/−	495 (226)	Ref.	0.16	495 (87)	Ref.	0.08
	−/−	680 (327)	1.13 (0.95–1.34)		680 (90)	0.77 (0.57–1.03)	

CNV: copy number variant; HR: hazard ratio; CI: confidence interval

a Presented effect estimates and statistical significance are based on univariable Cox proportional hazard regression;

b P for trend for independent rs2978974 SNP at the 8q24.3 locus is 0.75 and 0.83 in relation to NMIBC recurrence and progression, respectively;

c P for trend for causal risk variant (rs17863783) at the 2q37.1 locus is 0.36 and 0.85 in relation to NMIBC recurrence and progression, respectively;

d rs1495741: tag SNP for *NAT2* acetylation status (GG  =  rapid, AG  =  intermediate, AA  =  slow)

#### Association of UBC susceptibility loci with disease progression

**Figure 1:**
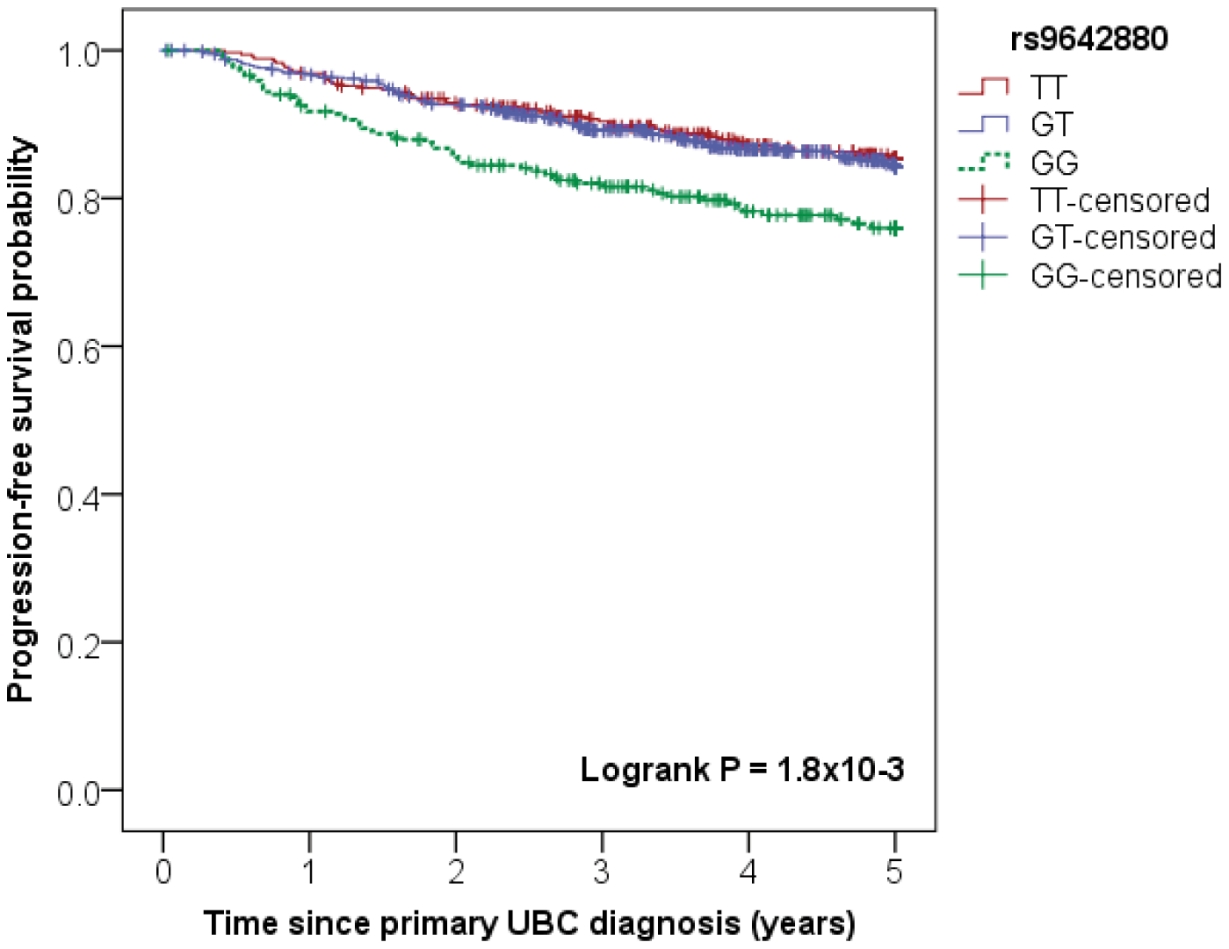
Association between rs9642880 (*MYC* locus) and progression-free survival in NMIBC patients. Kaplan-Meier survival plot showing association between rs9642880 genotype and progression-free survival (PFS) of non-muscle invasive bladder cancer (NMIBC) patients.

Median time at risk for progression of the 1,269 NMIBC patients was 4.9 years. 195 NMIBC patients (Kaplan-Meier 5-year risk: 17.2%) experienced disease progression during the first five years after the primary UBC diagnosis. One of the 12 genetic variants, *i.e.* rs9642880 at the *MYC* (v-myc avian myelocytomatosis viral oncogene homolog) locus showed a statistically significant association with the risk of disease progression (P for trend  =  2.6×10^−3^). The genotype-specific results suggest a recessive mode of action (GG vs. TT: HR  =  1.81 (95% confidence interval (CI): 1.23–2.66)), with no evidence of a difference in progression risk between GT heterozygotes and TT homozygotes (see Table 3 and Figure 1). With a stricter progression definition, *i.e.*, transition from NMIBC (Ta/T1/CIS) to muscle-invasive disease (≥T2) (60 events within 5 years of diagnosis; Kaplan-Meier 5-year risk: 5.5%), the association of rs9642880 became even more pronounced and increased risk of progression was observed among both heterozygous and homozygous carriers of the G allele (GT vs TT: HR  =  1.49 (95% CI: 0.72–3.08) and GG vs. TT: HR  =  3.15 (95% CI: 1.50–6.61), P for trend =  1.32×10^−3^). In addition, the stricter progression definition revealed nominal evidence for increased risk of progression among carriers of the rs710521 [G] allele (AG vs. AA: HR  =  1.52 (95% CI: 0.89–2.61) and GG vs. AA: HR  =  2.84 (95% CI: 1.24–6.51), P for trend  =  0.01) in NMIBC patients. None of the other risk variants were nominally or statistically significantly associated with time to progression.

#### Subgroup analysis according to tumor aggressiveness

For several of the susceptibility loci, the etiologic link with bladder cancer was previously reported to be specific for (or most prominent among) the group with low or high risk of progression. Therefore, we also evaluated whether the association with disease recurrence and progression for the 12 genetic variants varies between these NMIBC subgroups. We adjusted for any remaining treatment variability within the two NMIBC subgroups. After restriction to NMIBC with known type of initial treatment, 672 were classified as ‘at low risk of progression’, and 534 as ‘at high risk of progression’, while for 12 cases aggressiveness could not be determined. Five-year risk of recurrence is 48% and 52% among low and high risk cases, respectively (indicating that tumor aggressiveness is not a good classifier for the risk of recurrence). With respect to progression, five-year risk varies between 9% among low risk and 26% among high risk cases. The stratified analysis did not reveal statistically significant associations in either of the subgroups, not for recurrence and not for progression; some suggestive findings of (borderline) nominal significance were found (see Table S2).

#### Effect modification by smoking status

Because of their involvement in the detoxification of xenobiotic and carcinogenic substances (including constituents of cigarette smoke, the main UBC risk factor), we also evaluated the association of *NAT2* acetylation status, *GSTM1* CNV, and of the rs11892031 SNP (in the UDP glucuronosyltransferase 1 family, polypeptide A complex locus (*UGT1A*) cluster) with NMIBC prognosis according to smoking status (see Table 4). Data on self-reported smoking status and type of initial treatment was available for 1,035 NMIBC patients (852 and 183 ever and never (cigarette) smokers, respectively). The analyses revealed some evidence for an elevated risk of disease recurrence in *NAT2* slow acetylators compared to patients with an intermediate or rapid acetylation status specifically among ever smokers (rs1495741 AA vs. AG/GG: HR_adj._  =  1.29 (1.04–1.61), P for trend  =  0.02). Besides, among never smokers, *NAT2* slow acetylation status was found to correlate with a decreased risk of disease progression compared to intermediate/rapid acetylators (HR_adj._  =  0.42 (0.19–0.93), P for trend  =  0.03). No evidence for effect modification by smoking status was found for the association between *GSTM1* CNV or rs11892031 and NMIBC prognosis.

**Table 4:** Association of selected UBC susceptibility variants with NMIBC recurrence and progression by smoking status.

		Disease recurrence^a^						Disease progression^a^					
		Never (cigarette) smokers (N = 183)			Ever (cigarette) smokers (N = 852)			Never (cigarette) smokers (N = 183)			Ever (cigarette) smokers (N = 852)		
SNP/CNV	Genotype	N (n events)	HR (95% CI)	P trend	N (n events)	HR (95% CI)	P trend	N (n events)	HR (95% CI)	P trend	N (n events)	HR (95% CI)	P trend
rs1495741^b^	GG/AG	60 (23)	ref.	0.63	302 (119)	ref.	0.02	60 (13)	ref.	0.03	302 (34)	ref.	0.27
	AA	123 (58)	1.13 (0.69–1.83)		549 (258)	1.29 (1.04–1.61)		123 (13)	0.42 (0.19–0.93)		549 (78)	1.26 (0.84–1.89)	
*GSTM1* del.	+/+ and +/−	74 (31)	ref.	0.25	335 (141)	ref.	0.42	74 (12)	ref.	0.86	335 (48)	ref.	0.29
	−/−	96 (44)	1.32 (0.83–2.11)		457 (203)	1.09 (0.88–1.36)		96 (12)	0.93 (0.42–2.09)		457 (50)	0.81 (0.54–1.20)	
rs11892031	AA	154 (68)	ref.	0.88	712 (316)	ref.	0.95	154 (23)	ref.	0.60	712 (91)	ref.	0.37
	AC	27 (12)	0.97 (0.52–1.80)		138 (59)	0.95 (0.72–1.26)		27 (3)	0.77 (0.23–2.58)		138 (21)	1.28 (0.79–2.07)	
	CC	2 (1)	1.88 (0.25–14)		2 (2)	2.08 (0.52–8.40)		2 (0)	C.E.		2 (0)	C.E.	
rs798766^c^	CC	119 (39)	ref.	**2.7**×**10^−5^**	497 (216)	ref.	0.75	119 (11)	ref.	0.09	497 (68)	ref.	0.93
	CT	59 (39)	**2.71 (1.73**–**4.24)**		304 (139)	1.07 (0.86–1.32)		59 (14)	2.50 (1.14–5.52)		304 (38)	1.05 (0.70–1.56)	
	TT	5 (3)	**2.43 (0.73**–**8.05)**		50 (22)	0.98 (0.63–1.52)		5 (1)	1.14 (0.15–9.02)		50 (6)	0.86 (0.37–1.99)	

CNV: copy number variant; del.  =  deletion; C.E.: converging error; HR: hazard ratio; CI: confidence interval

a Presented effect estimates and statistical significance are based on multivariable Cox proportional hazard regression analyses with adjustment for treatment (TURT + both adjuvant i.v. CT and IT *vs.* TURT + adjuvant i.v. IT *vs.* TURT + adjuvant i.v. CT *vs.* TURT only (± one direct p.o. i.v. CT instillation));

b rs1495741: tag SNP for *NAT2* acetylation status (GG  =  rapid, AG  =  intermediate, AA  =  slow);

c based on exploratory analysis

**Figure 2:**
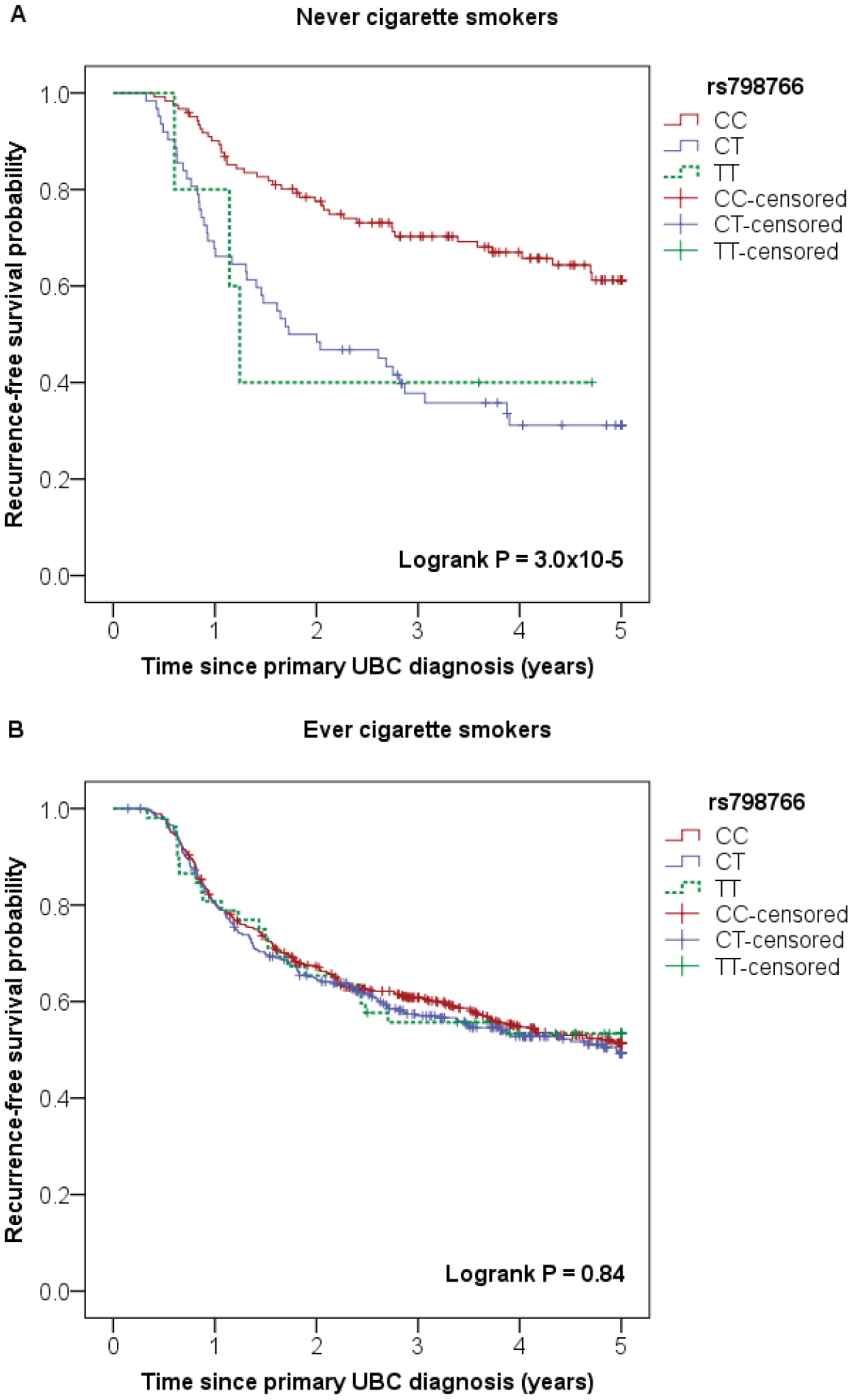
Association between rs798766 (*TACC3/FGFR3* locus) and recurrence-free survival in NMIBC patients by smoking status. Kaplan-Meier survival plots showing association between rs798766 genotype and recurrence-free survival (RFS) in **A**) never cigarette smokers (Logrank P  =  3.0×10^−5^) and **B**) ever cigarette smokers (Logrank P  =  0.84) with non-muscle invasive bladder cancer (NMIBC).

Interestingly, an exploratory analysis for the other susceptibility loci indicated association of rs798766 at the 4p16.3 (*TACC3-FGFR3*) locus with the risk of disease recurrence specifically among never smoking NMIBC patients (never cigarette smokers: CT vs. CC: HR_adj._  =  2.71 (95% CI: 1.73–4.24) and TT vs. CC: HR_adj._  =  2.43 (95% CI: 0.73–8.05), P for trend  =  2.7×10^−5^; ever cigarette smokers: CT vs. CC: HR_adj._  =  1.07 (95% CI: 0.86–1.32) and TT vs. CC: HR_adj._  =  0.98 (95% CI: 0.63–1.52), P for trend  =  0.75) (see Table 4 and Figure 2).

#### Prognostic analysis of genetic risk score

In addition to evaluation of the prognostic relevance of the UBC susceptibility variants at the single-SNP level, we assessed their cumulative (additive) effect on NMIBC prognosis by testing association with the overall number of UBC risk alleles (*i.e.*, the genetic risk score). After restriction to patients with complete genotype data for all 12 variants and known treatment type, 872 NMIBC patients (390 recurrence and 117 progression events) were included. The majority of ineligible patients for this analysis were due to a missing genotype for the rs2736098 SNP (N = 279: not included in Centaurus assay [22]) or the *GSTM1* CNV (N = 94; insufficient DNA amount or CNV analysis failed). This analysis indicated a trend towards a slightly worse RFS (HR_adj._  =  1.05 (95% CI: 1.00–1.10, P for trend  =  0.05) with each extra risk allele carried. This translates into a ∼1,3-fold and 1,6-fold increased risk of recurrence among carriers of 5 and 10 risk alleles (compared to patients with zero UBC risk alleles), respectively. No association was observed between the genetic risk score and PFS among NMIBC patients (HR_adj._  =  0.99 (95% CI: 0.91–1.07), P for trend  =  0.77).

### Muscle-invasive and metastatic bladder cancer (MIBC)

The Nijmegen Bladder Cancer Study contains 275 MIBC patients. Two patients were excluded from the analysis because they were previously or simultaneously diagnosed with cancer of the upper urinary tract.

#### Association of UBC susceptibility loci with overall survival

Median time at risk for overall death of the 273 MIBC patients was 6.8 (IQR: 3.1–11)) years. During the first 5 years after the primary diagnosis, 81 of the MIBC patients (Kaplan-Meier 5-year risk: 29.9%) died. None of the 12 variants evaluated was found to be associated with OS at the Bonferroni-adjusted statistical significance level (see Table 5). *GSTM1* CNV showed a statistically significant association with OS at the nominal P<0.05: HR  =  1.70 (95% CI: 1.04–2.79) for the patients with the null genotype versus all others. The association lost nominal significance however, after adjustment for the survival difference between patients with extended/metastasized and localized cancer (see Table 5). Because of the small sample size, no subgroup analyses were performed.

**Table 5:** Association of confirmed UBC susceptibility variants with overall mortality among MIBC patients.

			Unadjusted (N = 273)		Adjusted^a^ (N = 273)	
SNP/CNV	Genotype	N (n events)	HR (95% CI)	P trend	HR (95% CI)	P trend
rs9642880	TT	63 (18)	Ref.	0.69	Ref.	0.56
	GT	139 (39)	0.94 (0.54–1.64)		0.86 (0.49–1.50)	
	GG	70 (23)	1.12 (0.61–2.08)		1.18 (0.64–2.18)	
rs710521	AA	152 (50)	Ref.	0.16	Ref.	0.12
	AG	101 (27)	0.77 (0.48–1.23)		0.88 (0.55–1.41)	
	GG	20 (4)	0.59 (0.21–1.63)		0.52 (0.19–1.45)	
rs2294008^b^	CC	79 (18)	Ref.	0.22	Ref.	0.25
	CT	133 (43)	1.45 (0.84–2.52)		1.22 (0.70–2.13)	
	TT	61 (20)	1.48 (0.78–2.79)		1.45 (0.77–2.74)	
rs798766	CC	178 (49)	Ref.	0.21	Ref.	0.28
	CT	85 (27)	1.13 (0.71–1.80)		1.03 (0.65–1.65)	
	TT	10 (5)	2.14 (0.85–5.37)		2.41 (0.96–6.05)	
rs401681	CC	100 (24)	Ref.	0.13	Ref.	0.29
	CT	114 (36)	1.29 (0.77–2.17)		1.23 (0.73–2.06)	
	TT	59 (21)	1.56 (0.87–2.79)		1.39 (0.76–2.45)	
rs2736098	GG	99 (31)	Ref.	0.10	Ref.	0.35
	AG	80 (15)	0.54 (0.29–0.99)		0.64 (0.34–1.19)	
	AA	18 (4)	0.68 (0.24–1.93)		0.95 (0.33–2.72)	
rs11892031^c^	AA	243 (69)	Ref.	0.20	Ref.	0.20
	AC	30 (12)	1.50 (0.81–2.76)		1.49 (0.81–2.75)	
	CC	0 (0)	NA		NA	
rs8102137	TT	107 (34)	Ref.	0.49	Ref.	0.57
	CT	130 (38)	0.92 (0.58–1.45)		0.93 (0.59–1.48)	
	CC	36 (9)	0.77 (0.37–1.61)		0.81 (0.39–1.69)	
rs1014971	AA	123 (34)	Ref.	0.97	Ref.	0.92
	AG	124 (41)	1.20 (0.76–1.89)		1.05 (0.66–1.66)	
	GG	26 (6)	0.79 (0.33–1.89)		0.87 (0.37–2.08)	
rs1058396	GG	78 (28)	Ref.	0.29	Ref.	0.45
	AG	137 (37)	0.73 (0.44–1.19)		0.69 (0.42–1.13)	
	AA	58 (16)	0.75 (0.41–1.39)		0.85 (0.46–1.57)	
rs1495741^d^	GG/AG	92 (32)	Ref.	0.17	Ref.	0.09
	AA	181 (49)	0.73 (0.47–1.14)		0.68 (0.43–1.06)	
*GSTM1* deletion	+/+ and +/−	104 (23)	Ref.	0.03	Ref.	0.09
	−/−	146 (51)	1.70 (1.04–2.79)		1.53 (0.93–2.51)	

CNV: copy number variant; HR: hazard ratio; CI: confidence interval;

a with adjustment for extended/metastasized (*i.e.*, primary stage T4(b) ór any T with N+/N≥1 and/or M1) *vs.* localized disease (*i.e.*, primary stage T2-T4a with N0/NX and M0/MX) in multivariable Cox proportional hazard regression analyses;

b P for trend (unadjusted) for independent rs2978974 SNP at the 8q24.3 locus is 0.16;

c P for trend (unadjusted) for causal risk variant (rs17863783) at the 2q37.1 locus is 0.92;

d rs1495741: tag SNP for *NAT2* acetylation status (GG  =  rapid, AG  =  intermediate, AA  =  slow)

### Regional association analysis

The evaluation of the association of (measured and imputed) genetic variants within a 200 kb region centered on each of the 10 GWAS-identified susceptibility SNPs did not reveal an association at the statistical significance threshold P<1×10^−4^ (on average ∼500 variants in 200 kb region) for any of the genetic markers in relation to the endpoints RFS and PFS in the overall NMIBC subgroup (see Figure S1 and S2) and OS in the MIBC subgroup.

For the *MYC* locus, rs9642880 shows the second strongest association signal; rs10094872 shows a slightly stronger association signal with the risk of NMIBC progression (P = 1.8×10^−3^; r^2^ between the two SNPs based on 1000G Pilot 1 CEU data  =  0.54). After adjusting for the effect of rs9642880, the association of rs10094872 lost strength in terms of the HR and statistical significance (P = 0.25). The same holds for the association of rs9642880 (P = 0.37) conditional on the effect of rs10098472, indicating that both variants represent the same association signal.

## Discussion

There is increasing evidence that the same genes or genetic variants could be implicated in both cancer predisposition, disease prognosis, and treatment response. So far, this interrelatedness at the genetic level is particularly observed for genes involved in well-known oncogenic pathways such as xenobiotic metabolism, DNA repair, and cell cycle control. [29], [30] In addition, several GWAS-identified cancer susceptibility loci (several with yet unknown mechanism) have been found to play a role in cancer prognosis. [14]–[21] This is the first comprehensive evaluation of the prognostic relevance of all confirmed UBC susceptibility loci.

Based on this study, for only one of the investigated UBC susceptibility loci an association with disease prognosis was identified which passed the multiple testing threshold. This main finding indicated an effect of the rs9642880 SNP at the *MYC* locus on PFS in the overall group of NMIBC patients. Patients with the rs9642880 GG genotype experienced an increased risk of disease progression compared to patients with the GT or TT genotype. Various etiological studies (including the initial GWAS) have indicated consistently that the risk of UBC is increased for carriers of the rs9642880[T] allele, which was most pronounced for low grade Ta tumors. [23], [31]–[35] This etiologic link of rs9642880[T] with a less aggressive type of UBC could explain the decreased risk of progression in carriers of this allele. However, we do observe the decreased risk of progression in both the low and high risk of progression subgroups. This implies that within these risk strata rs9642880[T] confers an additional beneficial influence on the clinical disease course. Studies in other cancer types show an association for other (independent) SNPs in the 8q24 chromosomal region with disease aggressiveness and/or clinical outcome. [14], [36]–[41] This strengthens the importance of unraveling the mystery of this 8q24 locus, a so-called gene desert, which seems to be involved in the susceptibility and clinical disease course of multiple cancer types. In addition to validation in an independent patient population, it requires further investigation whether the association that we found is mediated through a long-range, regulatory effect on the 30 kb upstream located *MYC* proto-oncogene, or through another biological mechanism. [42], [43]

Previously, in one of the publications based on our GWAS for bladder cancer risk, we described association of the rs798766[T] allele (*TACC3/FGFR3* locus) with an increased risk of disease recurrence among low-stage low-grade NMIBC cases. [21] We do observe a similar trend in this paper (low risk of progression subgroup: CT vs. CC: HR_adj._  =  1.27 (95% CI: 1.00–1.61), and TT vs. CC: HR_adj._  =  1.18 (95% CI: 0.71–1.94), P for trend  =  0.09). However the association is less pronounced and does not pass the significance threshold. This difference in effect estimates may be explained by the fact that this analysis and the previous GWAS analysis are based on only a partly overlapping patient series and use a slightly different recurrence definition. Also, in our current evaluation, we adjusted for the independent effect of treatment among the strata according to tumor aggressiveness. Based on an exploratory analysis, we found indications that the association between rs798766 and NMIBC recurrence might be mediated by smoking status, but this has to be confirmed by other studies.

For several other susceptibility loci, our study provides suggestive evidence for an association with UBC prognosis, especially in subgroup analyses, which could reflect the different molecular pathways that play a role in different disease subtypes. We presented the association results in this paper, but do not discuss them in further detail here as these findings first require replication in independent UBC patient series. Evaluation among the MIBC subgroup is hampered by the relatively small sample size. Future analyses should be performed in larger patient series that allow analyses in relevant subgroups with respect to disease stage and treatment.

Our prognostic evaluation based on a cumulative genetic risk score suggests that UBC risk loci might collectively influence NMIBC recurrence. This finding may be due to a cumulative effect of multiple small increases in recurrence risk conferred by several of the susceptibility variants.

Where classification of NMIBC patients into two risk strata with respect to tumor aggressiveness resulted in reasonable discrimination in the risk of progression in our cohort, it appeared to be a poor classifier for disease recurrence. Lack of data on tumor size and exact tumor number (two important recurrence predictors in the EORTC risk model but characteristics that are poorly documented in medical charts) for a large proportion of patients limited us in our possibilities to improve prognostic risk classification. Risk group stratification according to European Association of Urology (EAU) guidelines (*i.e.*, low, intermediate, high risk) resulted in too low event numbers to perform valid prognostic evaluation. [44] Consequently, the ‘low risk of progression’ subgroup contains a mix of low and intermediate risk cases, which could have diminished the power to detect subgroup-specific associations. However, we adjusted for remaining treatment variability among both risk strata, and thereby expect to have (indirectly) corrected for the correlated prognostic variables incorporated in the EORTC scoring system. In contrast to data underlying the EORTC prediction model, this study focused on primary bladder cancers (no prior recurrences), which most likely diminished variability in recurrence risk due to lack of cases at the higher end of the spectrum.

Major strengths of this study are the population-based nature, the relatively large sample size, and the medical file review of all patients. A weakness of the study is the prevalent sampling frame of part of the study cohort, which might have led to a relatively healthy study population with possible implications for the generalizability of our study findings to all UBC patients. We expect that this selection is negligible in NMIBC patients, especially with respect to the endpoint recurrence. The relatively high five-year OS in the MIBC subgroup (∼70%) could reflect the effect of prevalent case sampling. The selection of patients with a less severe disease course could have resulted in some bias in the effect size measures.

With exception of our finding for the *MYC* locus, this study provides only suggestive evidence that genetic loci involved in UBC etiology have prognostic relevance. Replication studies in independent UBC series are necessary to confirm our findings, and should include evaluation among (disease) subgroups. Elucidation of the causal mechanism in functional analyses will further our understanding of the disease, might eventually point the way to potential new therapeutic targets, and could aid in further improvement of prognostic risk discrimination.

### Note

After acceptance of this research paper, Figueroa *et al*. [48] reported on two new GWAS-identified UBC susceptibility loci. We included the association results of these two UBC risk variants (*i.e.*, rs10936599 (3q26.2) and rs907611 (11p15.5)) with disease prognosis in File S1. For both variants, we did not observe association with clinical outcome that passed the Bonferroni-adjusted statistical significance threshold, in both the NMIBC and MIBC subgroup.

## Supporting Information

Figure S1 Regional association plots for NMIBC recurrence of 200 kb region centered on 10 GWAS-identified susceptibility SNPs. **A–J**) In the above plots, directly genotyped and imputed single-nucleotide polymorphisms (SNPs) distributed in a 200 kb region centered on each of the 10 respective GWAS-identified urinary bladder cancer (UBC) susceptibility SNPs are depicted by filled circles. For each SNP, the chromosomal location (NCBI Build 36/hg18) is shown on the x-axis and the significance level for association with non-muscle invasive bladder cancer (NMIBC) recurrence is indicated by a -log_10_ P-value on the left y-axis. In each plot, the GWAS-identified UBC susceptibility SNP at the respective locus is represented by a purple diamond. Local linkage disequilibrium (LD) structure is reflected by the plotted estimated recombination rates from 1000 Genomes Pilot June 2010 CEU (light blue line, right y-axis). The level of correlation (LD) of the UBC susceptibility SNP to other SNPs at the locus (pair wise r^2^ values) are indicated by a color range from dark blue to red (see legend). SNPs with missing LD information are shown in grey. Below the graph, gene annotations are shown as horizontal dark blue lines. Regional association plots are shown for the **A**) 2q37.1 locus (rs11892031): most significant association for rs116323695 (P = 2.35E-02), **B**) 3q28 locus (rs710521): most significant association for rs3773928 (P = 2.03E-03), **C**) 4p16.3 locus (rs798766): most significant association for rs73081713 (P = 5.09E-03), **D**) 5p15.33 locus (rs2736098): most significant association for rs6420010 (P = 6.55E-03), **E**) 5p15.33 locus (rs401681): most significant association for rs116612249 (P = 8.29E-03), **F**) 8q24.3 locus (rs2294008): most significant association for rs118159558 (P = 2.33E-03), **G**) 8q24.21 locus (rs9642880): most significant association for rs80238840 (P = 7.65E-03), **H**) 18q12.3 locus (rs1058396): most significant association for rs8088466 (P = 7.72E-03), **I**) 19q12 locus (rs8102137): most significant association for rs117125197 (P = 6.16E-02), **J**) 22q13.1 locus (rs1014971): most significant association for rs7584 (P = 1.3E-02).
(ZIP)

Figure S2 Regional association plots for NMIBC progression of 200 kb region centered on 10 GWAS-identified susceptibility SNPs. **A–J**) In the above plots, directly genotyped and imputed single-nucleotide polymorphisms (SNPs) distributed in a 200 kb region centered on each of the 10 respective GWAS-identified urinary bladder cancer (UBC) susceptibility SNPs are depicted by filled circles. For each SNP, the chromosomal location (NCBI Build 36/hg18) is shown on the x-axis and the significance level for association with non-muscle invasive bladder cancer (NMIBC) progression is indicated by a -log_10_ P-value on the left y-axis. In each plot, the GWAS-identified UBC susceptibility SNP at the respective locus is represented by a purple diamond. Local linkage disequilibrium (LD) structure is reflected by the plotted estimated recombination rates from 1000 Genomes Pilot June 2010 CEU (light blue line, right y-axis). The level of correlation (LD) of the UBC susceptibility SNP to other SNPs at the locus (pair wise r^2^ values) are indicated by a color range from dark blue to red (see legend). SNPs with missing LD information are shown in grey. Below the graph, gene annotations are shown as horizontal dark blue lines. Regional association plots are shown for the **A**) 2q37.1 locus (rs11892031): most significant association for rs13009407 (P = 3.24E-03), **B**) 3q28 locus (rs710521): most significant association for rs76380205 (P = 5.07E-03), **C**) 4p16.3 locus (rs798766): most significant association for rs73081713 (P = 9.29E-03), **D**) 5p15.33 locus (rs2736098): most significant association for rs246993 (P = 2.49E-03), **E**) 5p15.33 locus (rs401681): most significant association for rs246993 (P = 2.49E-03), **F**) 8q24.3 locus (rs2294008): most significant association for rs73716487 (P = 3.26E-04), **G**) 8q24.21 locus (rs9642880): most significant association for rs10094872 (P = 1.83E-03), **H**) 18q12.3 locus (rs1058396): most significant association for rs12454702 (P = 8.85E-03), **I**) 19q12 locus (rs8102137): most significant association for rs16963425 (P = 5.46E-02), **J**) 22q13.1 locus (rs1014971): most significant association for rs7289061 (P = 2.25E-04).
(ZIP)

File S1 Association of two newly confirmed GWAS-identified UBC susceptibility variants with UBC prognosis.
(DOC)

Table S1 Descriptive characteristics of excluded NMIBC patients with immediate radical cystectomy (N = 19).
(DOCX)

Table S2 Association of UBC susceptibility variants with NMIBC recurrence and progression by tumor aggressiveness.
(DOCX)

Text S1 Detailed description of prognostic endpoint definitions.
(DOC)
