# Supplementary material for: Prognostic Relevance of Urinary Bladder Cancer Susceptibility Loci
Source: PLoS One. 2014 Feb 25;9(2):e89164. doi: 10.1371/journal.pone.0089164 (PMC3934869; doi:10.1371/journal.pone.0089164)
Supplement: File S1 — Association of two newly confirmed GWAS-identified UBC susceptibility variants with UBC prognosis. (DOC) [file pone.0089164.s003.doc]

**Supporting File S1.** Association of two newly confirmed GWAS-identified UBC susceptibility variants with UBC prognosis.

**Table S1.1.** Two newly identified and extensively replicated germline UBC susceptibility loci.

| **Locus** | **Gene region** | **SNP** | **Risk allele** | **Allelic OR** | **Risk allele frequency^a^** | **Study type** | **Reference** |
| --- | --- | --- | --- | --- | --- | --- | --- |
| 3q26.2 | *TERC, ACTRT3, MYNN, LRRC34* | rs10936599 | C | 1.18 | 0.76 | GWAS | [1] |
| 11p15.5 | *LSP1* | rs907611 | A | 1.15 | 0.31 | GWAS | [1] |

OR: odds ratio; *TERC*: telomerase RNA component; *ACTRT3*: actin-related protein T3.; *MYNN*: myoneurin; *LRRC34*: leucine rich repeat containing 34; *LSP1*: lymphocyte-specific protein 1

^a^ risk allele frequency as published in GWAS paper

**Table S1.2.** Association of two newly confirmed UBC susceptibility variants with NMIBC recurrence and progression

|  |  | **Disease recurrence (N=1,269)^a^** | | | **Disease progression (N=1,269)^a^** | | |
| --- | --- | --- | --- | --- | --- | --- | --- |
| **SNP** | **Genotype** | **N (n events)** | **HR (95% CI)** | **P trend** | **N (n events)** | **HR (95% CI)** | **P trend** |
| rs10936599 | CC | 754 (361) | Ref. | 0.74 | 754 (113) | Ref. | 0.74 |
|  | CT | 448 (212) | 1.01 (0.85-1.20) |  | 448 (73) | 1.12 (0.83-1.50) |  |
|  | TT | 67 (28) | 0.88 (0.60-1.29) |  | 67 (9) | 0.91 (0.46-1.79) |  |
| rs907611 | GG | 566 (271) | Ref. | 0.76 | 566 (93) | Ref. | 0.25 |
|  | AG | 554 (253) | 0.96 (0.81-1.13) |  | 554 (81) | 0.88 (0.65-1.19) |  |
|  | AA | 145 (74) | 1.10 (0.85-1.42) |  | 145 (19) | 0.78 (0.48-1.28) |  |

HR: hazard ratio; CI: confidence interval

^a^ Presented effect estimates and statistical significance are based on univariable Cox proportional hazard regression;

**Table S1.3.** Association of two newly confirmed UBC susceptibility variants with NMIBC recurrence and progression by tumor aggressiveness

|  |  | **Disease recurrence^a^** | | | | | | **Disease progression^a^** | | | | | |
| --- | --- | --- | --- | --- | --- | --- | --- | --- | --- | --- | --- | --- | --- |
|  |  | **Low risk^b^ (N=672)** | | | **High risk^c^ (N=534)** | | | **Low risk^b^ (N=672)** | | | **High risk^c^ (N=534)** | | |
| **SNP** | **Genotype** | **N (n events)** | **HR (95% CI)** | **P trend** | **N (n events)** | **HR (95% CI)** | **P trend** | **N (n events)** | **HR (95% CI)** | **P trend** | **N (n events)** | **HR (95% CI)** | **P trend** |
| rs10936599 | CC | 409 (182) | Ref. | 0.90 | 312 (151) | Ref. | 0.96 | 409 (32) | Ref. | 0.94 | 312(71) | Ref. | 0.58 |
|  | CT | 231 (100) | 0.99 (0.77-1.26) |  | 190 (93) | 1.03 (0.80-1.34) |  | 231 (20) | 1.19 (0.68-2.08) |  | 190 (48) | 1.17 (0.81-1.69) |  |
|  | TT | 32 (14) | 0.98 (0.57-1.70) |  | 32 (13) | 0.95 (0.54-1.68) |  | 32 (1) | 0.43 (0.06-3.12) |  | 32 (7) | 1.00 (0.46-2.19) |  |
| rs907611 | GG | 278 (127) | Ref. | 0.59 | 268 (130) | Ref. | 0.32 | 278 (22) | Ref. | 0.97 | 268 (66) | Ref. | 0.46 |
|  | AG | 309 (130) | 0.89 (0.69-1.13) |  | 213 (97) | 0.98 (0.75-1.27) |  | 309 (23) | 0.91 (0.51-1.63) |  | 213 (50) | 0.98 (0.68-1.41) |  |
|  | AA | 83 (38) | 0.97 (0.67-1.39) |  | 51 (28) | 1.38 (0.92-2.08) |  | 83 (7) | 1.05 (0.45-2.46) |  | 51 (9) | 0.72 (0.36-1.44) |  |

HR: hazard ratio; CI: confidence interval

^a^ Presented effect estimates and statistical significance are based on multivariable Cox proportional hazard regression analyses with adjustment for treatment (TURT + both adjuvant i.v. CT and IT *vs.* TURT + adjuvant i.v. IT *vs.* TURT + adjuvant i.v. CT *vs.* TURT only (± one direct p.o. i.v. CT instillation));

^b^ Low risk of progression: Ta low grade tumors;

^c^ High risk of progression: all other NMIBC tumors

**Table S1.4.** Association of two newly confirmed UBC susceptibility variants with overall mortality among MIBC patients

|  |  |  | **Unadjusted (N=273)** | | **Adjusted^a^ (N=273)** | |
| --- | --- | --- | --- | --- | --- | --- |
| **SNP** | **Genotype** | **N (n events)** | **HR (95% CI)** | **P trend** | **HR (95% CI)** | **P trend** |
| rs10936599 | CC | 164 (59) | Ref. | 0.02 | Ref. | 0.17 |
|  | CT | 19 (17) | 0.46 (0.27-0.79) |  | 0.54 (0.32-0.94) |  |
|  | TT | 18 (5) | 0.72 (0.29-1.79) |  | 1.15 (0.45-2.91) |  |
| rs907611 | GG | 137 (39) | Ref. | 0.63 | Ref. | 0.77 |
|  | AG | 111 (33) | 1.06 (0.67-1.69) |  | 1.04 (0.65-1.65) |  |
|  | AA | 21 (7) | 1.23 (0.55-2.74) |  | 1.13 (0.51-2.53) |  |

HR: hazard ratio; CI: confidence interval;

^a^ with adjustment for extended/metastasized (*i.e.*, primary stage T4(b) ór any T with N+/N≥1 and/or M1) *vs.* localized disease (*i.e.*, primary stage T2-T4a with N0/NX and M0/MX) in multivariable Cox proportional hazard regression analyses;

**References**

1. Figueroa JD, Ye Y, Siddig A, Garcia-Closas M, Chatterjee, N et al. (2013) Genome-wide association study identifies multiple loci

associated with bladder cancer risk. Hum Mol Genet doi:10.1093/hmg/ddt519.
