# Supplementary material for: Prognostic Relevance of Urinary Bladder Cancer Susceptibility Loci
Source: PLoS One. 2014 Feb 25;9(2):e89164. doi: 10.1371/journal.pone.0089164 (PMC3934869; doi:10.1371/journal.pone.0089164)
Supplement: Table S1 — Descriptive characteristics of excluded NMIBC patients with immediate radical cystectomy (N = 19). (DOCX) [file pone.0089164.s004.docx]

| **N (%)** |  | **N=19** |
| --- | --- | --- |
| Male gender |  | 17 (89) |
| Median age, yrs (range) |  | 61 (39-75) |
| Smoking status | Never cigarette smoker | 3 (16) |
|  | Ever cigarette smoker | 12 (63) |
|  | Unknown | 4 (21) |
| Tumor stage | 0a | 2 (11) |
|  | 0is | 0 (0) |
|  | I | 17 (89) |
| Concomitant CIS | No | 10 (53) |
|  | Yes | 9 (47) |
| Tumor grade | Low grade | 0 (0) |
|  | High grade | 19 (100) |
| Tumor aggressiveness | Low risk of progression | 0 (0) |
|  | High risk of progression | 19 (100) |
| Tumor histology | UCC | 19 (100) |
|  | Other | 0 (0) |
| Tumor size | <3 cm | 0 (0) |
|  | ≥3 cm | 1 (5) |
|  | Unknown | 18 (95) |
| Tumor focality | Solitary | 2 (11) |
|  | Multifocal | 15 (79) |
|  | Unknown | 2 (11) |
| Recurrence (within 5 yrs) | No | 19 (100) |
|  | Yes | 0 (0) |
| Progression (within 5 yrs) | No | 17 (89) |
|  | Yes | 2 (11) |
| Progression to MIBC (within 5 yrs) | No | 17 (89) |
|  | Yes | 2 (11) |

**Table S1.** Descriptive characteristics of excluded NMIBC patients with immediate radical cystectomy (N=19).

CIS: carcinoma in situ; UCC: urothelial cell carcinoma; NMIBC: non-muscle invasive bladder cancer; MIBC: muscle-invasive bladder cancer
