# Supplementary material for: Prognostic Relevance of Urinary Bladder Cancer Susceptibility Loci
Source: PLoS One. 2014 Feb 25;9(2):e89164. doi: 10.1371/journal.pone.0089164 (PMC3934869; doi:10.1371/journal.pone.0089164)
Supplement: Table S2 — Association of UBC susceptibility variants with NMIBC recurrence and progression by tumor aggressiveness. (DOCX) [file pone.0089164.s005.docx]

**Table S2.** Association of UBC susceptibility variants with NMIBC recurrence and progression by tumor aggressiveness

|  |  | **Disease recurrence^a^** | | | | | | **Disease progression^a^** | | | | | |
| --- | --- | --- | --- | --- | --- | --- | --- | --- | --- | --- | --- | --- | --- |
|  |  | **Low risk^b^ (N=672)** | | | **High risk^c^ (N=534)** | | | **Low risk^b^ (N=672)** | | | **High risk^c^ (N=534)** | | |
| **SNP/CNV** | **Genotype** | **N (n events)** | **HR (95% CI)** | **P trend** | **N (n events)** | **HR (95% CI)** | **P trend** | **N (n events)** | **HR (95% CI)** | **P trend** | **N (n events)** | **HR (95% CI)** | **P trend** |
| rs9642880 | TT | 216 (104) | ref. | 0.45 | 121 (62) | ref. | 0.59 | 216 (18) | ref. | 0.49 | 121 (23) | ref. | 0.04 |
|  | GT | 341 (137) | 0.75 (0.58-0.97) |  | 268 (123) | 0.99 (0.72-1.34) |  | 341 (21) | 0.72 (0.38-1.35) |  | 268 (60) | 1.20 (0.74-1.94) |  |
|  | GG | 113 (55) | 0.96 (0.69-1.33) |  | 140 (68) | 1.10 (0.77-1.56) |  | 113 (14) | 1.41 (0.70-2.84) |  | 140 (41) | 1.69 (1.01-2.83) |  |
| rs710521 | AA | 392 (178) | ref. | 0.20 | 305 (155) | ref. | 0.56 | 392 (33) | ref. | 0.25 | 305 (68) | ref. | 0.08 |
|  | AG | 244 (107) | 0.95 (0.74-1.20) |  | 199 (84) | 0.79 (0.61-1.04) |  | 244 (19) | 0.89 (0.51-1.57) |  | 199 (45) | 1.01 (0.69-1.47) |  |
|  | GG | 36 (11) | 0.62 (0.34-1.14) |  | 29 (17) | 1.28 (0.78-2.12) |  | 36 (1) | 0.27 (0.04-1.99) |  | 29 (13) | 2.41 (1.32-4.38) |  |
| rs2294008 | CC | 171 (70) | ref. | 0.11 | 133 (63) | ref. | 0.47 | 171 (21) | ref. | 0.51 | 133 (35) | ref. | 0.42 |
|  | CT | 372 (162) | 1.09 (0.83-1.45) |  | 298 (147) | 1.06 (0.79-1.42) |  | 372 (30) | 1.17 (0.60-2.28) |  | 298 (69) | 0.87 (0.58-1.31) |  |
|  | TT | 128 (64) | 1.33 (0.95-1.87) |  | 103 (47) | 0.86 (0.59-1.25) |  | 128 (11) | 1.31 (0.58-2.98) |  | 103 (22) | 0.81 (0.47-1.38) |  |
| rs798766 | CC | 378 (154) | ref. | 0.09 | 334 (157) | ref. | 0.57 | 378 (29) | ref. | 1.00 | 334 (77) | ref. | 0.75 |
|  | CT | 256 (125) | 1.27 (1.00-1.61) |  | 172 (86) | 1.07 (0.82-1.39) |  | 256 (22) | 1.12 (0.65-1.96) |  | 172 (43) | 1.15 (0.79-1.68) |  |
|  | TT | 37 (17) | 1.18 (0.71-1.94) |  | 28 (14) | 1.11 (0.64-1.93) |  | 37 (2) | 0.71 (0.17-2.96) |  | 28 (6) | 0.89 (0.39-2.06) |  |
| rs401681 | CC | 237 (116) | ref. | 0.36 | 185 (85) | ref. | 0.68 | 237 (26) | ref. | 0.06 | 185 (45) | ref. | 0.96 |
|  | CT | 327 (133) | 0.79 (0.62-1.01) |  | 268 (132) | 0.99 (0.75-1.30) |  | 327 (21) | 0.59 (0.33-1.05) |  | 268 (60) | 0.87 (0.59-1.28) |  |
|  | TT | 108 (47) | 0.94 (0.67-1.32) |  | 80 (40) | 1.11 (0.76-1.62) |  | 108 (6) | 0.51 (0.21-1.24) |  | 80 (21) | 1.05 (0.63-1.77) |  |
| rs2736098 | GG | 259 (103) | ref. | 0.11 | 205 (98) | ref. | 0.42 | 259 (14) | ref. | 0.06 | 205 (44) | ref. | 0.85 |
|  | AG | 226 (104) | 1.20 (0.92-1.58) |  | 162 (78) | 0.94 (0.70-1.27) |  | 226 (19) | 1.52 (0.76-3.04) |  | 162 (39) | 1.04 (0.67-1.60) |  |
|  | AA | 57 (28) | 1.33 (0.87-2.02) |  | 40 (16) | 0.81 (0.47-1.37) |  | 57 (7) | 2.41 (0.97-5.98) |  | 40 (9) | 1.05 (0.51-2.16) |  |
| rs11892031 | AA | 565 (246) | ref. | 0.55 | 445 (215) | ref. | 0.86 | 565 (42) | ref. | 0.36 | 445 (105) | ref. | 0.67 |
|  | AC | 103 (47) | 1.02 (0.75-1.40) |  | 89 (42) | 0.97 (0.69-1.36) |  | 103 (11) | 1.48 (0.76-2.89) |  | 89 (21) | 1.11 (0.69-1.79) |  |
|  | CC | 4 (3) | 2.20 (0.70-6.86) |  | 0 (0) | - |  | 4 (0) | C.E. |  | 0 (0) | - |  |
| rs8102137 | TT | 301 (132) | ref. | 0.71 | 210 (104) | ref. | 0.85 | 301 (16) | ref. | 0.12 | 210 (53) | ref. | 0.71 |
|  | CT | 309 (136) | 0.96 (0.75-1.22) |  | 243 (114) | 0.97 (0.74-1.27) |  | 309 (32) | 1.91 (1.05-3.49) |  | 243 (51) | 0.77 (0.52-1.14) |  |
|  | CC | 55 (23) | 0.94 (0.60-1.47) |  | 80 (39) | 1.07 (0.74-1.55) |  | 55 (4) | 1.37 (0.46-4.11) |  | 80 (21) | 1.02 (0.62-1.70) |  |
| rs1014971 | AA | 307 (136) | ref. | 0.63 | 230 (116) | ref. | 0.43 | 307 (22) | ref. | 0.40 | 230 (53) | ref. | 0.78 |
|  | AG | 306 (134) | 0.93 (0.74-1.19) |  | 253 (120) | 1.00 (0.77-1.29) |  | 306 (24) | 1.09 (0.61-1.94) |  | 253 (64) | 1.11 (0.77-1.60) |  |
|  | GG | 59 (26) | 0.95 (0.62-1.44) |  | 50 (21) | 0.77 (0.49-1.23) |  | 59 (7) | 1.55 (0.66-3.66) |  | 50 (9) | 0.75 (0.37-1.51) |  |
| rs1058396 | GG | 202 (89) | ref. | 0.98 | 161 (66) | ref. | 0.04 | 202 (12) | ref. | 0.29 | 161 (36) | ref. | 0.37 |
|  | AG | 329 (142) | 0.93 (0.71-1.21) |  | 262 (129) | 1.28 (0.95-1.73) |  | 329 (29) | 1.56 (0.80-3.07) |  | 262 (58) | 0.99 (0.65-1.51) |  |
|  | AA | 140 (65) | 1.02 (0.74-1.40) |  | 111 (62) | 1.43 (1.01-2.02) |  | 140 (12) | 1.51 (0.68-3.36) |  | 111 (32) | 1.26 (0.78-2.03) |  |
| rs1495741^d^ | GG/AG | 246 (102) | ref. | 0.29 | 182 (83) | ref. | 0.36 | 246 (26) | ref. | 0.79 | 182 (46) | ref. | 0.43 |
|  | AA | 425 (194) | 1.14 (0.90-1.45) |  | 352 (147) | 1.13 (0.87-1.47) |  | 425 (34) | 1.08 (0.61-1.89) |  | 352 (80) | 0.86 (0.60-1.24) |  |
| *GSTM1* del. | +/+ and +/- | 264 (119) | ref. | 0.98 | 208 (93) | ref. | 0.14 | 264 (26) | ref. | 0.10 | 208 (56) | ref. | 0.21 |
|  | -/- | 358 (155) | 1.00 (0.79-1.27) |  | 285 (141) | 1.22 (0.94-1.59) |  | 358 (21) | 0.62 (0.35-1.10) |  | 285 (58) | 0.79 (0.55-1.14) |  |

CNV: copy number variant; del. = deletion; HR: hazard ratio; CI: confidence interval; CE: converging error

^a^ Presented effect estimates and statistical significance are based on multivariable Cox proportional hazard regression analyses with adjustment for treatment (TURT + both adjuvant i.v. CT and IT *vs.* TURT + adjuvant i.v. IT *vs.* TURT + adjuvant i.v. CT *vs.* TURT only (± one direct p.o. i.v. CT instillation));

^b^ Low risk of progression: Ta low grade tumors;

^c^ High risk of progression: all other NMIBC tumors;

^d^ rs1495741: tag SNP for *NAT2* acetylation status (GG = rapid, AG = intermediate, AA = slow)
