# Supplementary material for: Prognostic Relevance of Urinary Bladder Cancer Susceptibility Loci
Source: PLoS One. 2014 Feb 25;9(2):e89164. doi: 10.1371/journal.pone.0089164 (PMC3934869; doi:10.1371/journal.pone.0089164)
Supplement: Text S1 — Detailed description of prognostic endpoint definitions. (DOC) [file pone.0089164.s006.doc]

# Supporting Text S1

**Endpoint definitions**

The date of first recurrence was defined as the first date after the initial diagnosis of NMIBC at which there was histological confirmation of a new bladder or prostatic urethra tumor with at least:

- one intermediate tumor-negative urethrocystoscopy (UCS), ór
- two previous surgical resection attempts for the primary bladder tumor (*e.g.,* after a TURT and a restaging TURT the next tumor counts as a recurrence; a negative UCS is not necessary).

One (endoscopic) coagulation/fulguration of a small tumor (*i.e.*, no histological confirmation) did not count as a recurrence. Every second coagulation/fulguration did count as a recurrence.

The date of first progression was defined as the first date after the initial diagnosis of NMIBC at which at least one of the following five types of progression occurred:

- transition from a low-grade (*i.e.*, WHO 1973 differentiation grade 1 or 2, WHO/ISUP 2004 low grade, ór Malmström (modified Bergkvist) grade 1 or 2a) primary bladder tumor to a high-grade (*i.e.*, WHO 1973 differentiation grade 3, WHO/ISUP 2004 high grade, ór Malmström (modified Bergkvist) grade 2b or 3) UBC recurrence.
- an increase in T stage (*i.e.*, from primary Ta/CIS to a recurrence with ≥T1 ór from primary T1 to a recurrence with ≥T2)
- an increase in N stage (*i.e.*, from NX/N0 to ≥N1)
- an increase in M stage (*i.e.*, from MX/M0 to M1)
- cystectomy for therapy-resistant or “uncontrollable” disease
